# The Mashujaa Network: youth-driven resilience for reproductive health and safe abortion in conflict-affected communities

**DOI:** 10.3389/frph.2026.1814179

**Published:** 2026-06-02

**Authors:** Amos M. Makelele, Mike Mpoyi, Jean-Claude Mulunda, Nadia Lobo Jive, Michee Kanda Sanza, Beniel Ulrich Agossou, Simon Binezero Mambo

**Affiliations:** 1 Youth Alliance for Reproductive Health (YARH), Goma, Democratic Republic of Congo; 2 Ipas, Kinshasa, Democratic Republic of Congo; 3 Centre ODAS, Abidjan, Côte d'Ivoire

**Keywords:** conflict-affected communities, Mashujaa Network, reproductive health, safe abortion, youth-driven resilience

## Abstract

Sexual and reproductive health (SRH) and rights are fundamental to individual well-being and development. Yet, significant global gaps in access to SRH information and services threaten the lives and health of millions of individuals and their families. Protecting the health of vulnerable populations in conflict zones is a critical public health priority. There is a significant lack of evidence on effective approaches to delivering comprehensive sexual and reproductive health (SRH) interventions in these settings. In the Democratic Republic of Congo (DRC), where unsafe abortion is a leading cause of maternal mortality, the Mashujaa Youth Referral Network was developed as an innovative, youth-led model to bridge this gap. By training young individuals as Mashujaa Champions, the network provides a trusted, community-based pathway for advocacy, peer education, and direct referral to safe abortion and other essential SRH services. This participatory approach demonstrates the profound potential of locally led initiatives to not only expand access to life-saving care and reduce stigma, but also to empower young people to become agents of change. Scaling this proven framework offers a sustainable solution for strengthening SRH resilience and reducing preventable maternal deaths in humanitarian contexts.

## Sexual and reproductive health in crisis settings – a forgotten priority

Sexual and reproductive health (SRH) and rights are essential for individual well-being and progress. However, a widespread lack of access to SRH information and services around the world endangers the lives of millions of people and their families (1). This is particularly critical in humanitarian contexts, where an estimated 32 million women and girls of reproductive age are living in dire need of these services (2).

Humanitarian crises increase the risk of poor SRH outcomes due to factors like reduced access to services and supplies, damaged health facilities, and a rise in sexual violence. These factors have implications across the life cycle (1–3). Armed conflict disrupts the entire healthcare system, leading to a long-lasting breakdown in the provision of family planning, safe motherhood services, and protection from gender-based violence (GBV) as individuals struggle to survive (4).

The Democratic Republic of Congo has endured decades of protracted conflict, leaving millions of people displaced and the health system in chronic disrepair (5, 6). In humanitarian responses, SRH services are consistently underprioritized, with severely limited access to contraception and safe abortion care, especially in camps for internally displaced persons (IDPs). In these settings, women and girls face elevated risks of unintended pregnancies, sexually transmitted infections (STIs), and complications from unsafe abortions (5).

The DRC also records some of the highest rates of sexual violence globally and is frequently cited as the “rape capital of the world”. In conflict-affected provinces, women and girls are subjected to mass rape, sexual slavery, and forced pregnancies, with thousands of cases reported annually (6). These violations directly contribute to high levels of unintended pregnancies, many of which end in unsafe abortions—one of the leading causes of maternal morbidity and mortality in these contexts (5).

Although comprehensive abortion care has been identified as a priority in the 2010 Inter-Agency Field Manual on SRH in Humanitarian Settings, reproductive health for conflict-affected populations has long been a neglected public health issue (3, 7). Despite the DRC's restrictive abortion law, thousands of women continue to obtain abortions every year in Kinshasa, of which many are performed unsafely, resulting in severe complications and sometimes death, In Kinshasa, the annual abortion rate is estimated at 56 to 105 per 1,000 women, with approximately 400 unsafe abortions occurring daily. While services may sometimes be provided, their availability and quality are inconsistent across IDP settings. This gap is particularly concerning because unsafe abortion remains a major cause of maternal mortality globally, with its prevalence likely increasing in humanitarian settings as health systems collapse. Yet, despite the fact that abortion procedures can be safely performed in health centres and even through mobile clinics by mid-level providers without sophisticated equipment, stakeholders in these contexts often fail to recognize abortion care as a critical health priority (8).

The DRC has ratified the African Union's Maputo Protocol, which legalizes abortion in cases of sexual assault, rape, incest, foetal anomalies, and when the pregnancy endangers the mother's life or mental and physical health (9). However, access to legal abortion remains severely limited due to inconsistent legal enforcement, slow integration of the protocol into national laws, and crippling social stigma against women who seek or have had abortions. This does not prevent women from having abortions, but it pushes many toward dangerous, unsafe methods. While unsafe abortion causes an estimated 5.1%–17.2% of maternal deaths in Sub-Saharan Africa, the burden is particularly severe in the Democratic Republic of the Congo (DRC). Each year, approximately 16,000 Congolese women die during their pregnancy, childbirth, or within six weeks after the end of their pregnancy (9). The situation is alarming in Eastern DRC, where around 27% of women report experiencing sexual assault in their lifetime, and 17% of those raped report becoming pregnant as a result. Among survivors who sought to terminate these pregnancies, a survey found that 65% relied on traditional methods that are often ineffective and potentially dangerous (9, 30).

Protecting the health of the most vulnerable populations in the Democratic Republic of the Congo (DRC) is a critical public health priority (5). The devastating impact of unsafe abortion and limited access to reproductive healthcare which contributes to thousands of maternal deaths annually highlights a crucial gap in service provision. It is essential to provide comprehensive sexual and reproductive health (SRH) interventions to women affected by armed conflict, but there is a lack of evidence on effective approaches to delivering such interventions in conflict settings. This review synthesised the role of young people in advancing reproductive justice in Humanitarian crisis (10). To address access to safe abortion care in Humanitarian crisis, Youth Alliance for Reproductive Health (YARH) with the support of Ipas under the Makoki Ya Mwasi program developed the Mashujaa Youth Referral Network, an innovative youth-led model. This network serves as a direct intervention to bridge the service gap, placing young people at the center of advocacy, referrals, and service navigation to ensure that at-risk populations can access comprehensive sexual and reproductive health and rights (SRHR) services in conflict-affected communities.

This manuscript presents a descriptive case study based on a retrospective review of program monitoring data. The intervention period covered in this report spans from January 2022 to December 2024, focusing on conflict-affected communities in the North Kivu province of Eastern DRC. The Mashujaa Network was implemented by the Youth Alliance for Reproductive Health (YARH), which led community mobilization, peer education, and referral activities. Ipas DRC provided technical oversight, clinical training [including Values Clarification and Transformation (VCAT) workshops], and quality assurance for the sexual and reproductive health (SRH) services to which clients were referred.

## Mashujaa Network youth-led model for safe abortion care: the central role of community engagement

**Figure 1:**
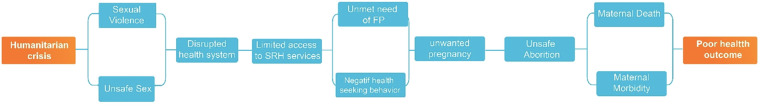
Pathway for impacts on women's SRH in humanitarian crisis.

Humanitarian crises create a complex web of barriers to essential healthcare. Health systems are often severely disrupted or completely collapse, leading to a lack of trained providers, medicine shortages, and inaccessibility of facilities (11). In the DRC and other conflict-affected regions, this breakdown is compounded by a range of social and logistical obstacles that push communities towards unsafe options and low utilisation of sexual and reproductive health services (5, 11) (Figure 1).

Previous studies have highlighted community-based interventions, particularly those led by community health workers (CHWs) and community members, as effective in increasing service utilization. CHWs, viewed as trusted members of their communities, have played a critical role in delivering interventions such as contraception provision and education on gender-based violence (10, 12). Community engagement emerged as a common facilitator, particularly through activities such as social mobilization, empowerment initiatives, and outreach strategies that helped build trust. Approaches frequently used included theatre and drama groups, as well as radio broadcast messages (10, 13).

Young people are uniquely positioned to communicate with their peers, creating a non-judgmental space for open dialogue about SRH issues (14). Peer-to-Peer Education Programs have shown that peer educators can effectively increase knowledge and uptake of services by leveraging existing social networks (15). In countries such as the DRC, organizations have trained community volunteers to serve as community-based distributors (CBDs) of contraceptives and other sexual and reproductive health (SRH) commodities. This approach helps bypass damaged infrastructure by bringing essential supplies directly to hard-to-reach populations, thereby ensuring continued access even in times of conflict (13, 14). Youth Referral Models don't just provide information; they actively link young people to care (16). This includes providing referrals to trusted, youth-friendly clinics and even accompanying them to appointments to overcome logistical and attitudinal barriers. Peer-to-peer networks and community health workers (CHWs) are essential in humanitarian settings as they serve as trusted, culturally sensitive links between vulnerable communities and life-saving sexual and reproductive health services (10, 12). By leveraging their position as respected community members, these advocates can effectively address barriers such as stigma, fear, and misinformation, which often prevent women from seeking care in formal health systems (13). They provide critical services like education on family planning and gender-based violence (GBV) and establish safe, non-judgmental referral pathways, thereby empowering individuals to make informed decisions and access comprehensive care that might otherwise be unavailable.

A major challenge is the presence of “false clinics” facilities that misrepresent themselves as providers of abortion care but instead aim to dissuade women from seeking legal, safe services (17). This disinformation preys on the vulnerability of women and girls in crisis. Additionally, conscientious objection by healthcare providers who refuse to offer services based on personal beliefs, as well as judgmental attitudes, can shame patients and act as a significant barrier, particularly for young people (18, 30). These factors create a climate of fear and distrust, forcing many to resort to dangerous, unsafe abortion methods.

The Mashujaa Network (“brave” in Swahili) was established to address the gap in entry points to sexual and reproductive health (SRH) including access to safe abortion care in conflict zones (9, 19, 20). Unlike traditional, top-down interventions that often fail to meet the nuanced needs of affected communities, Mashujaa was designed by young people, for young people, enhancing its relevance, trust, and adaptability in fragile contexts. Launched in 2022, the initiative was developed by the Youth Alliance for Reproductive Health (YARH) with Ipas RDC which provides ongoing technical support (9, 21). The core of the Mashujaa approach is its participatory framework, which trains young individuals to become “Mashujaa Champions”. These champions are effective advocates for SRHR and are knowledgeable about key legal instruments like the Maputo Protocol, which guarantees women's health and reproductive rights.

**Figure 2:**
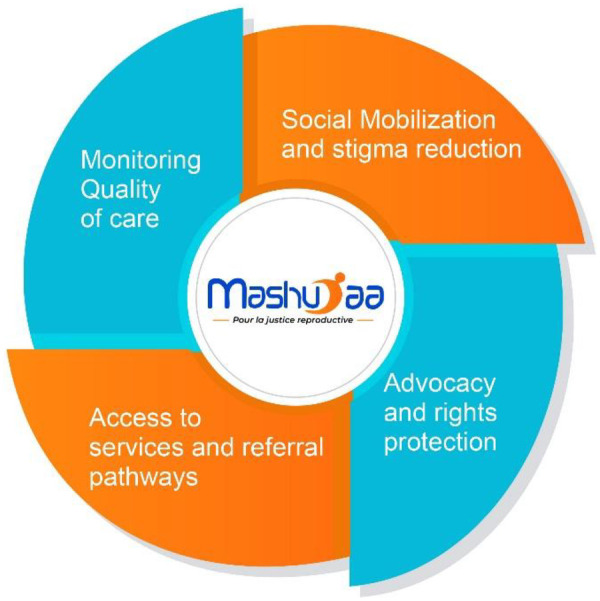
Mashujaa Network strategies.

The champions do more than just provide information; they actively guide and support young people through referral pathways, answer their questions, and work to reduce stigma around contraception and safe abortion. By collaborating with trained, youth-friendly service providers, the Mashujaa Network makes SRH services more accessible and trustworthy. This comprehensive, context-sensitive approach mobilizes local youth to directly improve health outcomes in humanitarian settings (Figure 2).

- Serving as a bridge between the legal rights guaranteed by the Maputo Protocol and the community. By “vulgarizing” the Protocol—a term used to mean making its complex legal language accessible and understandable to the general public, the network empowers individuals with knowledge of their rights to safe and legal abortion in cases of sexual assault, rape, and incest, in cases of fetal malformation, or where there is risk to a pregnant person's life or health (21) (PRB, 2018). This advocacy extends beyond simple awareness, as the network actively works to dismantle the barriers of stigma and misinformation, while also establishing a practical referral system that connects women in need with trained healthcare providers, ensuring that the progressive policies of the Maputo Protocol are not just words on a page, but a reality for women and girls on the ground.
- Reducing the stigma associated with abortion by promoting a new approach that prioritizes the dignity and respect of women (3, 22, 23). Rather than treating abortion as a crime or a moral failing, the network's work is designed to reframe it as a healthcare matter. This involves shifting attitudes among community members and healthcare professionals alike, ensuring that women seeking care are met with support, not judgment (23). By embedding these values at the core of their policy advocacy, the Mashujaa Network aims to create an environment where women feel safe to access the legal and medical services they need, transforming the conversation around reproductive health from one of shame to one of compassion and care.
- Connects women and girls to abortion services through a comprehensive community-based approach (24). The network, which includes, Mashujaa, Community members, healthcare providers, displaced people and identifying individuals in need during outreach services and mobile clinics, often in humanitarian settings. Mashujaa Network provides essential information and then refers clients to a pre-established network of trained providers or to mobile clinics for care (24, 25). Beyond just making a connection, the Mashujaa network provides crucial support by sometimes accompanying women to their appointments and following up afterward to ensure they received the care they needed. This entire process, from initial contact to follow-up, allows them to evaluate the quality of care and ensure it is safe, respectful, and aligns with the new national guidelines.
- The Mashujaa Network uses technology to make safe abortion care more accessible, particularly in challenging environments. By leveraging telemedicine, they offer a safe and private channel for individuals to access information and guidance on self-managed abortion (26). This approach is crucial in conflict zones or remote areas where in-person clinics might be dangerous or simply unavailable. Through digital tools like a chatbot Nurse Nisa and Hesperian Health guides mobile app, the network provides women and girls with accurate, evidence-based health information, giving them the power to make informed decisions about their bodies in a confidential way (27, 28). This digital strategy ensures that the right to safe care is not limited by geography, security risks, or the stigma of in-person interactions.

## From barriers to bridges: Mashujaa Network delivers life-saving care through community engagement

Since its implementation in 2022, the Mashujaa Network's observed program outcomes have served as a powerful testament to its decentralized, survivor-centered model. Based on the retrospective review of our implementation data, a cornerstone of this success is the training of 75 youth champions under the Ipas' Makoki Ya Mwasi program and the Quality Innovation Challenge, supported by the David and Lucile Packard Foundation.

These youth underwent intensive workshops led by Ipas RDC technical experts, focusing on Values Clarification and Transformation (VCAT), community mobilization strategies, and advocacy. The curriculum placed heavy emphasis on ethical considerations for patient privacy and the technical protocols required to safely handle clinical referrals in volatile settings (9, 15, 23). This investment in local capacity created a community-embedded workforce that engaged over 52,597 individuals in a conflict zone, demonstrating a remarkable ability to penetrate disrupted social networks (14, 19).

Within this outreach, program records indicate that approximately 15,000 women and girls accessed reproductive health data through private channels of information such as encrypted messaging and discreet peer-to-peer counseling to avoid the risks of public stigmatization. Most critically, the network facilitated the direct provision of safe abortion care to 1,956 women and girls, representing a high-impact intervention within the project scope that has directly averted life-threatening complications and reduced the long-term burden on the DRC's fragile health system (20, 21). This tangible reduction in maternal morbidity and mortality highlights the program's success in turning advocacy into life-saving action. The reported decrease in the demand for post-abortion care is a key behavioral indicator, suggesting that more individuals are proactively accessing safe, preventative services, thereby avoiding the need for emergency care to manage complications from unsafe procedures (9, 27).

Beyond the numbers, the Mashujaa Network has created profound qualitative impacts by addressing underlying social and systemic barriers. Vulgarizing the Maputo Protocol transforms complex legal language into accessible knowledge and empowers communities to claim their rights (21). This gives women and girls the confidence and knowledge to assert their rights in a society where those rights are often ignored or denied. The program directly confronts the social and moral stigmas that have long been weaponized against women, reframing abortion as a legitimate healthcare matter. This shift in attitudes among both community members and providers creates a virtuous cycle: as stigma decreases, more women feel safe seeking care, which in turn normalizes the service and further reduces stigma (18).

The Mashujaa Network is effective because it operates as a complete ecosystem of support, not a single intervention. It bridges individuals in crisis with trained professionals and directly counters the disinformation propagated by facilities that exploit vulnerable women and girls (24). By establishing a reliable referral system and sometimes even accompanying women to appointments, the network goes beyond simple information provision to address the practical and emotional barriers to care. This holistic approach ensures that no one is left to navigate the process alone. Finally, the strategic use of technology telemedicine through a chatbot and mobile applications has been a crucial innovation for humanitarian contexts. These tools provide a safe and private channel for communication, bypassing security risks and geographical constraints to deliver accurate, confidential information directly to those who need it most (27).

## Catalyzing change: scaling the Mashujaa Network for enduring resilience

The success of the Mashujaa approach in Eastern DRC demonstrates its potential for replication in other humanitarian settings. By positioning youth at the center of advocacy and service facilitation, the model strengthens SRH resilience and addresses preventable maternal deaths in fragile environments. Its effectiveness highlights the value of investing in local capacities and empowering communities to lead change within their own contexts. Adapting the Mashujaa framework to other crisis-affected regions requires a nuanced approach that prioritizes contextualization over simple replication. While the core principles of youth leadership and peer-to-peer engagement are powerful everywhere, their application must be sensitive to local cultural, legal, and social dynamics (14). For instance, the model's success in the DRC in making the Maputo Protocol's legal protections accessible (“vulgarizing” the Protocol) would need to be re-evaluated based on the specific legal frameworks of a new country. In settings with different legal frameworks on abortion, the network's focus might shift from legal education to a greater emphasis on confidential referrals and harm reduction. The Mashujaa model's strength is its ability to build trust through community-based distribution and youth-friendly referral pathways, which can be a template for addressing a new region's specific barriers, such as provider conscientious objection or the presence of “false clinics” (10, 16).

For the Mashujaa Network to achieve enduring sustainability, it must evolve from an effective project into a foundational component of the healthcare system. This requires strategic partnerships that transcend the initial collaboration with an international NGO like Ipas. Fostering strong ties with government health ministries ensures that the model can be integrated into national health strategies, potentially securing public funding and official recognition for its youth champions as a recognized tier of community health workers. Collaborating with diverse local community organizations and faith-based groups can also help to address the deep-seated stigma around reproductive health (13). These partnerships are essential to overcome the logistical and attitudinal barriers that plague fragile contexts, ensuring that the network's life-saving work is not reliant on short-term project cycles but is embedded in the long-term healthcare framework of the country.

To demonstrate its value and justify scaling, the Mashujaa model must be subjected to a rigorous longitudinal impact assessment. While the current quantitative results are compelling—reaching over 52,597 individuals and providing care to 1,956 women and girls, long-term data is crucial to prove that these changes are sustainable (24). Future research should evaluate the sustained effects on community perceptions of SRH and the Maputo Protocol. It should also track the long-term empowerment of the youth champions, assessing if their advocacy skills lead to continued leadership roles. By collecting this data, the Mashujaa Network can produce a body of evidence that moves beyond initial outputs and demonstrates a lasting transformation in both individual health outcomes and community resilience.

The lessons learned from the Mashujaa Network's success in the DRC provide a powerful foundation for evidence-based policy advocacy. The model's ability to reduce maternal mortality and overcome barriers through a participatory, youth-led framework is a compelling argument for prioritizing SRH services within humanitarian response plans. By leveraging the concrete data from its impact assessment, the network can advocate for specific policy changes, such as the official recognition and funding of youth health champions, or the integration of SRH education into national curricula. This advocacy can ensure that the progressive spirit of instruments like the Maputo Protocol is not just a legal abstraction but is actively protected and promoted in national health strategies and budgets, transforming the conversation around reproductive health from one of shame to one of compassion and care (23, 29).

## Conclusion: the Mashujaa Network's path to lasting change

The Mashujaa approach unequivocally proves that direct youth engagement in SRH mediation and advocacy is not merely a supplementary measure but a powerful and essential strategy for strengthening SRH resilience and reducing preventable maternal deaths in humanitarian contexts. This model offers a sustainable, community-driven solution by putting young people at the heart of the response, empowering them to address the long-neglected public health crisis of limited access to sexual and reproductive health (SRH) services. In humanitarian settings where formal health systems collapse and services are underprioritized, Mashujaa champions build trust and dismantle barriers like misinformation, stigma, and the disinformation from “false clinics”. The network's success in providing direct access to safe abortion care demonstrates that by bridging the gap between legal rights and on-the-ground reality, it is possible to save lives and avert thousands of severe complications. This model's ability to shift community perceptions and provide life-saving services ensures that vulnerable populations have access to a full range of care. As we look towards future humanitarian responses, the imperative to invest in and scale up such participatory models becomes paramount, as they foster healthier, more informed, and resilient communities capable of navigating the profound challenges posed by crises, ultimately upholding the fundamental right to health for all.

## Data Availability

The original contributions presented in the study are included in the article/Supplementary Material, further inquiries can be directed to the corresponding author.
